# Combined Ileoileal and Ileocolic Intussusception Secondary to Inflammatory Fibroid Polyp in an Adult: A Case Report

**DOI:** 10.3390/medicina58020310

**Published:** 2022-02-18

**Authors:** Hao-Tse Chiu, Hao Yen, Yu-Shiou Weng, Chao-Yang Chen, Kuan-Hsun Lin, Po-Huang Chen, Hong-Jie Jhou, Ta-Wei Pu

**Affiliations:** 1 Department of Surgery, Tri-Service General Hospital, National Defense Medical Center, Taipei 11490, Taiwan; haotsechiu@gmail.com (H.-T.C.); cartilage77@yahoo.com.tw (C.-Y.C.); dontchangela@gmail.com (K.-H.L.); 2 Department of Pathology, Tri-Service General Hospital, National Defense Medical Center, Taipei 11490, Taiwan; kastyplanet@gmail.com; 3 Department of Radiology, Tri-Service General Hospital, National Defense Medical Center, Taipei 11490, Taiwan; charlieboy2727@gmail.com; 4 Division of Colon and Rectal Surgery, Tri-Service General Hospital, National Defense Medical Center, Taipei 11490, Taiwan; 5 Division of Thoracic Surgery, Department of Surgery, Tri-Service General Hospital, National Defense Medical Center, Taipei 11490, Taiwan; 6 Department of Internal Medicine, Tri-Service General Hospital, National Defense Medical Center, Taipei 11490, Taiwan; chenpohuang@hotmail.com; 7 Department of Neurology, Changhua Christian Hospital, Changhua 50006, Taiwan; xsai4295@gmail.com; 8 School of Medicine, Kaohsiung Medical University, Kaohsiung 80708, Taiwan; 9 Department of Surgery, Division of Colon and Rectal Surgery, Songshan Branch, Tri-Service General Hospital, National Defense Medical Center, Taipei 10581, Taiwan

**Keywords:** ileoileal intussusception, ileocolic intussusception, adult intussusception, inflammatory fibroid polyp

## Abstract

Intestinal intussusception is relatively rare in adults and accounts for approximately 5% of intestinal obstruction. Intussusception is classified into subtypes according to the location, including ileoileal, ileocolic, ileo-ileocolic, colo-colic, jejuno-ileal, or jejuno-jejunal; the ileocolic type being the most common. However, intussusception of a combination of different subtypes has rarely been reported in the available literature. Abdominal computed tomography (CT) is the most accurate tool to evaluate intestinal intussusception. The pathological lead point in the intestine typically results in adult intussusception. Surgical intervention is usually adopted in cases of adult intussusception due to a high incidence of underlying bowel malignancy. An inflammatory fibroid polyp (IFP) is one of the uncommon benign neoplasms of the gastrointestinal (GI) system, which can result in intestinal intussusception. Herein, we present a case of a 50-year-old female with combined ileoileal and ileocolic intussusception, which was initially diagnosed by abdominal CT. Therefore, laparoscopic right hemicolectomy surgery was performed, confirming the final diagnosis as ileoileal and ileocolic intussusception secondary to IFP.

## 1. Introduction

Intestinal intussusception is relatively rare in adults and accounts for approximately 5% of small bowel obstruction. The clinical presentation of intussusception includes intermittent abdominal cramping pain, nausea, and vomiting.

The classification of intussusception depends on the location, including ileocolic, ileoileal, ileo-ileocolic, jejuno-jejunal, jejuno-ileal, or colo-colic. Abdominal computed tomography (CT) is the most accurate tool for evaluating intestinal intussusception. The ileocolic type is the most common. However, cases in which different types of intussusceptions occur simultaneously are rare. Adult intestinal intussusception typically occurs as a result of a pathologic lead point in the intestine, and surgical intervention is usually adopted in cases of adult intussusception, owing to the high bowel malignant rate. Inflammatory fibroid polyp (IFP), also known as a Vanek’s tumor, is one of the uncommon benign neoplasms of the gastrointestinal (GI) system [1], which can result in intestinal intussusception. Herein, we present a case of a 50-year-old female diagnosed with combined ileo-ileo and ileocolic intussusception, which was initially diagnosed by abdominal CT. Therefore, laparoscopic right hemicolectomy surgery was performed, which revealed that the final diagnosis was ileo-ileo and ileocolic intussusception secondary to IFP.

## 2. Case Report

**Figure 1:**
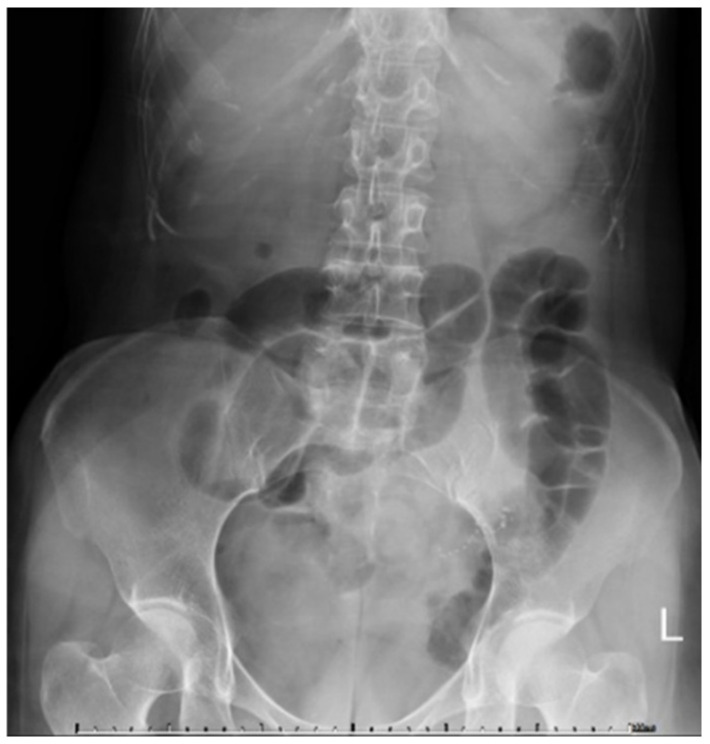
Plain abdominal radiograph revealed gaseous dilatation of the small bowel.

**Figure 2:**
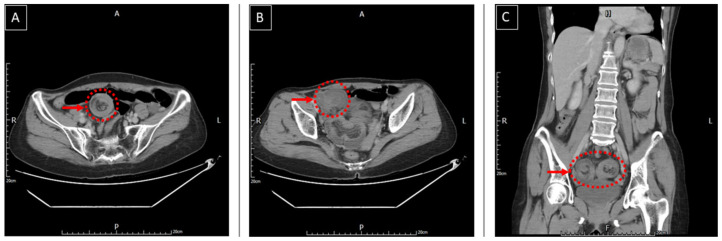
Abdominal computed tomography (CT); (**A**) The axial CT demonstrates an ileoileal intussusception with an accompanying complex of mesenteric fat and blood vessels, surrounded by the thick-walled intussuscipiens; (**B**) The axial CT demonstrates an ileocolic intussusception with associated bowel wall thickening and proximal small bowel dilatation; (**C**) The coronal CT demonstrates two target lesions with both ileoileal and ileocolic intussusception.

**Figure 3:**
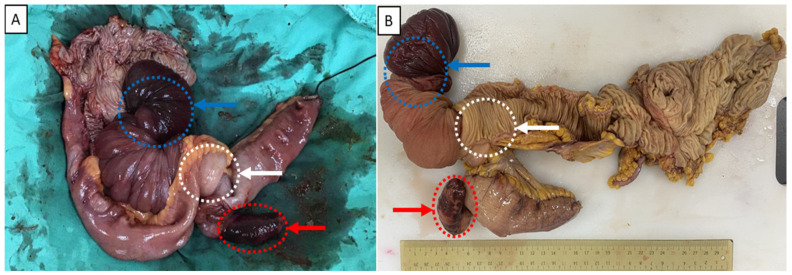
Macroscopic examination of the surgical specimen. (**A**) Macroscopic examination of the surgical specimen during surgery revealed a polypoid-shaped mass measuring 2.2 × 2.0 × 1.5 cm, arising from the mucosal surface at the distal ileum (Red arrow), which resulted in ileocolic (white circle) and ileoileal intussusceptions with an ischemic change at the terminal ileum (blue circle); (**B**) The surgical specimen was carefully incised transversely from the ascending colon to the cecum, and the ileocolic (white circle) and ileoileal intussusceptions were observed clearly. A polypoid-shaped mass measuring 2.2 × 2.0 × 1.5 cm, arising from the mucosal surface at the distal ileum (Red arrow).

**Figure 4:**
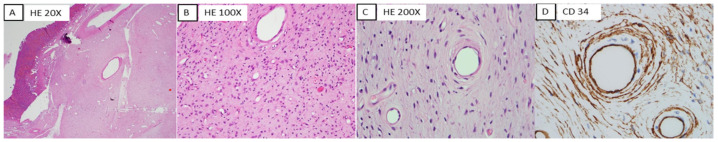
Histology findings of the tumor. (**A**) The inflammatory fibroid polyp (IFP) is a well-marginated but unencapsulated lesion arising from the submucosa of the ileum. (HE, 20×); (**B**) The IFP forms a fibrotic background with ovoid-to-spindle-shaped spindle cells mixed with eosinophils, lymphocytes, and plasma cells. (HE, 100×); (**C**) High magnification of the lesion shows that the cells form a whorled pattern, also called “onion skin,” which proliferated around the vessels. (HE, 200×); (**D**) The immunohistochemistry of CD34 is positive for the spindle cells and highlights whorled patterns around the vessels. (Immunohistochemical stain, 200×).

A 50-year-old woman visited our emergency department for worsening epigastralgia and intermittent vomiting for one day. She had epigastralgia and abdominal bloating for approximately 2 years without any regular medical record. She had no history of previous abdominal surgery or systemic disease. Physical examination revealed moderate tenderness around the epigastric region and increased muscle guarding and rebound tenderness. Abdominal auscultation revealed increased bowel sounds. Plain abdominal radiography showed gaseous dilatation of the small bowel (Figure 1). Abdominal CT revealed two target-like structures at the terminal ileum, with an ileoileal and an ileocolic intussusception, which cause obstruction with proximal small bowel dilatation (Figure 2). Laboratory tests conducted at the emergency department revealed the following: white blood cell count: 6.7 k/mm3 (4.2–10.3), hemoglobin: 12.4 g/dL (normal range: 12–16), platelet count: 207 k/mm3 (150–410), BUN: 5.1 mmol/L (2.5–6.1 mmol/L), SCr: 54 mmol/L (46–92 mmol/L, baseline), and C-reactive protein level: 1.0 mg/dL (<1.0 mg/dL). Based on the above findings, the diagnosis of ileoileal and ileocolic intussusception complicated with peritonitis was identified initially. Given that the severe abdominal tenderness was refractory to analgesic drugs, the surgeon performed urgent laparoscopic exploration. The intussuscipiens located in the cecum was visualized via the laparoscopy; thus, laparoscopic right hemicolectomy and end-to-end ileocolic anastomosis were performed due to a high suspicion of a malignant etiology. Macroscopic examination of the surgical specimen revealed a polypoid-shaped mass measuring 2.2 × 2.0 × 1.5 cm, arising from the mucosal surface at the distal ileum, which resulted in ileoileal and ileocolic intussusception with ischemic changes at the terminal ileum (Figure 3). Microscopic examination of the mass revealed a fibroid background with spindled cells intermixed with a few lymphocytes and eosinophils. The spindle cells also formed a whorled pattern around vessels in the lesion arising from the submucosa of the ileum, which is a characteristic that corresponds to IFP (Figure 4). The patient’s postoperative course was smooth and uneventful, and she was discharged on the 10th hospitalization day. She has been regularly followed up as an outpatient.

## 3. Discussion

Adult intussusception is an uncommon etiology of small bowel obstruction, which usually has a well-defined pathological abnormality of the leading point. In a recent meta-analysis by Hong et al. [2], the incidence of benign, malignant, and idiopathic etiologies accounted for 37.4%, 32.9%, and 15.1%, respectively. Most of the lead points in the small intestine comprise benign lesions, including benign neoplasms, inflammatory lesions, lipoma, Meckel’s diverticulum, and intestinal tubes [3,4]. Malignant lesions account for approximately 30% of cases of intestinal intussusception. In contrast, intussusception occurring in the colon are malignant in up to 66% of cases, including adenocarcinoma, leiomyosarcoma, lymphoma, and metastatic tumors [5]. The classification of intussusception depends on the location, including ileocolic, ileoileal, ileo-ileocolic, jejuno-jejunal, jejuno-ileal, or colo-colic. Generally, the ileocolic type is the most common. However, intussusception combining different subtypes has rarely been reported in the available literature. Abdominal CT is the most accurate tool to diagnose intussusception, but it does not precisely differentiate between the benign, malignant, or idiopathic etiology. Surgical intervention for adult intussusception involves radical resection, depending on its etiology and location. There is still a controversy on whether to perform intussusception reduction before radical resection. Resection without reduction is considered in adult intussusception when the bowel is inflamed, friable, or ischemic because of the high bowel malignant rate, in order to prevent intraluminal seeding, anastomotic complications of weakening during reduction, and the possibility of bowel perforation [6]. IFPs are rare benign neoplasms, and the most frequent location is the stomach and ileum. The pathogenetic mechanisms of IFP are almost sporadic and regarded as reactive lesions. However, activating gene mutations, including exon 12 and 18 and platelet-derived growth factor receptor alpha (PDGFRA), have been reported in sporadic cases in previous studies [7,8]. Most of the IFPs are asymptomatic, followed by abdominal pain, altered bowel habits, nausea, and vomiting, which depends on the size and location. IFP located in the ileum may manifest as bowel obstruction or intussusception [9]. The diagnosis of IFP depends on histopathologic evidence, with the unique characteristic of an onion skin appearance, the predominance of eosinophils, and CD34 and PDGFRA positivity, as shown by immunohistochemistry. Resection by laparoscopy or laparotomy is the recommended option for intussusception secondary to IFP to prevent the development of bowel ischemia, necrosis, and perforation. IFP secondary to intestinal intussusception, in the past decade, has been reviewed and published by Kao et al. in 2020 [10]. However, this is the first special case report concerning combined ileoileal and ileocolic intussusception caused by IFP, which is initially diagnosed as two target structures found by abdominal CT.

## 4. Conclusions

In conclusion, IFP-related intestinal intussusception is rare, especially a case combining ileoileal and ileocolic subtypes simultaneously. Abdominal CT is an accurate tool to diagnose intussusception. There is still no consensus regarding current treatment guidelines for adult intussusception. Due to the high malignant rate of the pathological leading point, surgical resection is regarded as the appropriate option. We described a rare case of combined ileoileal and ileocolic intussusception with peritonitis secondary to IFP in the distal ileum, which was successfully resected by laparoscopic right hemicolectomy.

## Data Availability

All data regarding the findings are available within the manuscript.
